# Innovative digital twin framework for early risk detection and personalized perinatal healthcare

**DOI:** 10.3389/fdgth.2026.1724029

**Published:** 2026-07-27

**Authors:** Mario Sanz Rodrigo, Virginia Anton Yuste, Diego Rivera, Jose Ignacio Moreno, Xavier Larriva Novo

**Affiliations:** Escuela Técnica Superior de Ingenieros de Telecomunicación (ETSIT), Universidad Politécnica de Madrid (UPM), Madrid, Spain

**Keywords:** digital twin, gestational diabetes, machine learning, maternal health, preeclampsia, pregnancy, synthetic data, wearables

## Abstract

**Introduction:**

Pregnancy-related complications such as gestational diabetes mellitus (GDM) and preeclampsia require timely identification, as initially low-risk pregnancies may develop clinically relevant risks during follow-up. Digital Health and Telemedicine can support remote monitoring, while Digital Twin (DT) technology offers a framework for integrating longitudinal patient data with predictive models. However, DT-based perinatal systems remain exploratory and require methodological validation before clinical use.

**Methods:**

This study presents a proof-of-concept DT framework for perinatal monitoring. Three data roles are distinguished: public benchmark datasets for model training and testing, synthetic patient records generated with Synthea to simulate prenatal consultations, and synthetic time-stamped wearable-like records generated with Gretel.ai to emulate repeated monitoring at the data-acquisition layer. Clinical and wearable-like streams were processed independently and combined only at the interpretation stage.

**Results:**

The maternal risk model performed better for low- and high-risk classes than for the intermediate class, supporting its use as a screening-oriented component rather than a definitive classifier. GDM models achieved high recall for the GDM class in both clinical and wearable-like configurations. By contrast, preeclampsia prediction showed limited performance, especially with wearable-accessible blood pressure variables alone; the clinical model improved the class profile but remained insufficient for clinical deployment. The simulated patient cases illustrate the DT workflow and the limitations of wearable-only inputs for complex obstetric risk stratification.

**Discussion:**

The proposed framework should be interpreted as a methodological prototype for organizing clinical and time-stamped wearable-like data within a perinatal DT. The results support combining episodic clinical information with repeated physiological monitoring, but also show that model calibration, class imbalance, richer clinical features, and validation with real longitudinal cohorts are needed before clinical decision-support use. Future work will focus on real-world validation, sequence-aware modelling, and integration with clinical workflows under medical supervision.

## 1 Introduction

Pregnancy monitoring requires the integration of episodic clinical information with physiological measurements collected between prenatal visits. This is particularly relevant for complications such as gestational diabetes mellitus (GDM) and preeclampsia, which may emerge or become clinically evident during follow-up. GDM is associated with maternal and neonatal complications and has a global prevalence that varies across populations and diagnostic criteria (1–3). Preeclampsia typically appears after the 20th week of gestation, is associated with hypertension and systemic maternal alterations, and remains a major contributor to maternal and perinatal morbidity and mortality (4–6). These conditions motivate monitoring approaches that combine clinical context with repeated physiological observations.

Digital Twins (DTs) provide a conceptual framework for organizing heterogeneous patient information into a computational representation of a physical system or process (7–9). In healthcare, DTs have been proposed for personalized medicine, remote monitoring, and decision-support workflows (10–12). Their use in maternal–fetal care remains exploratory, but recent work has investigated DT-based fetal and neonatal monitoring, multimodal pregnancy modelling, and risk-oriented maternal applications (13–18). In parallel, wearable and connected-monitoring technologies have shown potential for collecting physiological information outside clinical facilities, including blood pressure, ECG, temperature, heart-rate-related signals, and glucose-related measurements (19–23).

Despite these advances, several gaps remain for perinatal DT development. Clinical records and wearable measurements differ in temporal resolution, data quality, and clinical content; laboratory variables such as proteinuria, hemoglobin, HDL, or formal oral glucose tolerance testing cannot be replaced by wearable-accessible signals alone; and many predictive models are evaluated on task-specific tabular datasets rather than on validated longitudinal perinatal cohorts (24–31). Consequently, a perinatal DT prototype must make explicit which data are used for model development, which data are used for scenario simulation, and how the outputs of different prediction modules are brought together.

This study presents a proof-of-concept DT framework for pregnancy risk exploration. The end goal is not to deliver a clinically validated diagnostic system, but to evaluate whether heterogeneous data sources can be organized into a reproducible workflow that supports three functions: (i) development of separate prediction modules for general maternal risk, GDM, and preeclampsia using public benchmark datasets; (ii) post-training simulation of episodic clinical records and time-stamped wearable-like records using synthetic data; and (iii) interpretation-level integration of the three module outputs into a unified DT summary. The intended contribution is therefore methodological: to clarify the feasibility, role, and limitations of this architecture before validation with real longitudinal clinical cohorts.

## 2 Methods

The methodology follows the three functions defined in the Introduction. First, the DT architecture specifies how clinical and wearable-like inputs are processed and summarized. Second, the data layer separates public benchmark datasets used for model training and testing from synthetic Synthea and Gretel.ai records used for post-training scenario simulation. Third, the prediction layer defines record-level models for maternal risk, GDM, and preeclampsia, together with the interpretation-level procedure used to combine their outputs. The framework is a proof of concept; no real patient records or real wearable signals were used.

### 2.1 Digital twin system architecture

**Figure 1:**
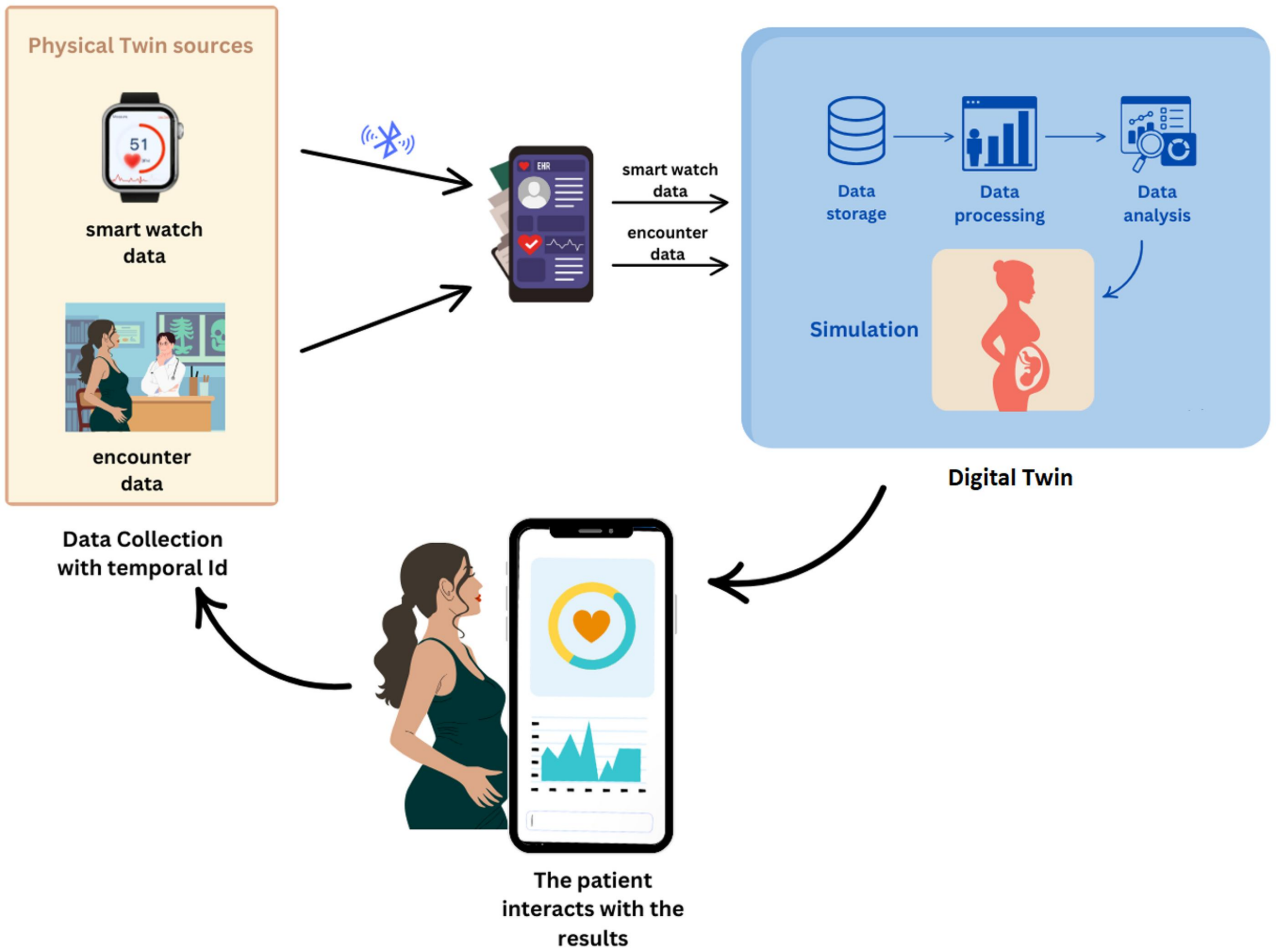
General architecture of the system based on a digital twin.

The system, shown in Figure 1, combines the DT paradigm with clinical and wearable-like data streams to support perinatal monitoring through four modules.

#### 2.1.1 Physical twin

The physical twin corresponds to the pregnant woman. In the proposed framework, her state is represented through two complementary data streams: episodic information collected during prenatal consultations and higher-frequency physiological measurements that emulate information obtainable from wearable devices.

#### 2.1.2 Data layer

Two data groups are considered. The first corresponds to time-stamped wearable-like measurements, including systolic and diastolic blood pressure, blood glucose, body temperature, and heart rate, simulated every eight hours. The second corresponds to clinical consultation data, including demographic information, vital signs, and laboratory measurements when available. These streams differ in both temporal resolution and clinical content; therefore, they are not merged at feature level during model development, and the wearable-like records are not treated as temporal sequences by the prediction models.

#### 2.1.3 Digital twin

The digital twin corresponds to the computational representation of the maternal state. It receives data from the physical twin, applies preprocessing and predictive models, and estimates three outputs: general maternal risk, GDM risk, and preeclampsia risk. In this proof-of-concept version, wearable-like and clinical data are processed independently and are brought together only during the final interpretation stage to support a unified risk summary. This final integration corresponds to an interpretation-level reporting layer rather than to a separate classifier trained on a joint multimodal dataset.

#### 2.1.4 Closed-loop interface

A patient-facing application was designed as the feedback layer between the digital and physical twins. Its intended function is to display risk trends, health indicators, and recommendations in an interpretable format. The recommendations are not autonomous clinical decisions; they are intended as supportive information that should be interpreted under medical supervision.

### 2.2 Data sources, data roles, and clinical parameters

The first methodological phase identified the clinical and biometric parameters used to represent the physical twin and to define the inputs of each risk model. Parameter selection was based on the project documentation, prenatal care protocols, and clinical relevance for the three target outputs: general maternal risk, GDM, and preeclampsia.

Table 1 summarizes the selected parameters, their clinical relevance, and the predictive models in which they are used. The table also reflects an important practical distinction: some variables can plausibly be monitored frequently through wearable or near-wearable devices, whereas others, such as proteinuria, hemoglobin, HDL, or formal oral glucose tolerance testing, require clinical or laboratory assessment.

**Table 1:** Clinical relevance of maternal parameters and their use in predictive models.

Parameter	Clinical relevance	Models used
Age	Risk factor for obstetric complications; advanced maternal age increases the probability of adverse outcomes.	Maternal Risk, GDM, Preeclampsia
BMI	Indicator of overweight or obesity; associated with insulin resistance and hypertensive complications.	GDM, Preeclampsia
Systolic BP	Sustained elevation suggests gestational hypertension and may indicate preeclampsia risk.	Maternal Risk, GDM, Preeclampsia
Diastolic BP	Complementary marker of hypertension; persistent increase is relevant for preeclampsia assessment.	Maternal Risk, GDM, Preeclampsia
Blood Glucose	Detects hyperglycemia and supports metabolic risk assessment.	Maternal Risk, GDM
Body Temperature	Identifies febrile states that may compromise pregnancy.	Maternal Risk
Heart Rate	Alterations may indicate maternal stress, anemia, or hemodynamic change.	Maternal Risk
HDL Levels	Lipid profile marker associated with metabolic syndrome and insulin resistance.	GDM
Hemoglobin Levels	Indicates anemia or altered oxygen-carrying capacity; included when available in clinical records.	GDM, Preeclampsia
Proteinuria	Core clinical marker for preeclampsia assessment and severity stratification.	Preeclampsia

BP, blood pressure; GDM, gestational diabetes mellitus.

### 2.3 Separation between model development and framework simulation

Table 2 summarizes the role of each data source. Performance metrics were computed on held-out or predefined test partitions from the original benchmark datasets. The synthetic Synthea and Gretel.ai datasets were used only after training to simulate the DT workflow, generate patient scenarios, and inspect record-level inference with clinical and wearable-like inputs. Descriptive summaries of raw input variables are therefore treated as methodological characterization of the simulated streams, not as standalone predictive results.

**Table 2:** Role of each data source in the proposed framework.

Data source	Use in this study	Purpose
Maternal Health Risk public dataset (32)	Model training and testing	Development of the general maternal risk classifier.
Gestational Diabetes public dataset (33)	Model training and testing	Development of the GDM classifier using clinically meaningful metabolic and hemodynamic variables.
Preeclampsia in pregnant women public dataset (34)	Model training and testing	Development of wearable-feature and clinical-feature preeclampsia MLP models using the predefined split available in the source dataset.
Synthea synthetic cohort	Post-training inference and scenario simulation	Generation of episodic prenatal consultation records and synthetic patient histories for DT workflow evaluation.
Gretel.ai time-stamped wearable-like records	Post-training inference and scenario simulation	Generation of repeated wearable-like vital-sign records with three daily records per patient for post-training DT simulation.

### 2.4 Synthetic data generation for digital twin simulation

Synthetic health data were used to evaluate the architecture in a controlled and privacy-preserving environment. No real patient records were accessed, processed, or included at any stage of the study. In addition, no real wearable measurements were collected; the term “wearable data” in this manuscript refers to synthetic time-stamped wearable-like records generated to emulate repeated physiological monitoring at the data-acquisition layer.

Synthetic data were not used as evidence of clinical validity. Their role was to test whether the framework could process heterogeneous data streams, preserve patient identifiers across clinical and repeated wearable-like records, and generate post-training scenario summaries. This distinction is important because synthetic data can support proof-of-concept engineering validation but cannot replace validation with real clinical cohorts.

#### 2.4.1 Clinical data generation with synthea

The synthetic patient cohort was generated using Synthea (35, 36). The cohort size used throughout the framework-level simulations was a target population of n=500 synthetic living female patients. Within this synthetic population, a risk-focused subset of 50 pregnancies with factors such as prediabetes or gestational hypertension was used for stress-testing and scenario analysis.

The Synthea execution was configured to make the generation process reproducible. The main parameters were: -p 500 for the target population, -g F to restrict the population to female patients, -a 16-55 for the simulated age interval, -s 500 as the population seed, -cs 200 as the clinician seed, -r 01012024 as the reference date, and -k keep.json as a custom inclusion module. The seed value -s 500 is a pseudo-random seed and not an additional sample-size parameter. The generated data were based on the careplans, encounters, patients, and observations files, which were subsequently processed to create synthetic patient datasets suitable for DT simulation.

Several filtering steps were applied to improve longitudinal consistency. Antenatal continuity was approximated by retaining synthetic pregnancies with sufficient follow-up across gestation, operationalized as patients with at least ten consultations in the simulated observation window. Additional filtering used plausible ranges for variables such as BMI, blood pressure, capillary glucose, and proteinuria. Values outside physiological ranges were removed from the normal scenario datasets but retained when clinically relevant in risk-focused scenario datasets, preserving abnormal patterns needed for stress-testing the framework.

From this process, three clinical scenario datasets were obtained:

- **MaternalRiskEncounter:** vital signs relevant to general maternal risk, including BMI, systolic and diastolic BP, temperature, heart rate, and glucose.
- **gdmEncounterDiabeticPatients:** metabolic variables relevant to GDM scenario analysis, including glucose, hemoglobin, HDL, and BMI when available.
- **preeclampsiaHypertensivePatients:** variables relevant to preeclampsia scenario analysis, including BP, proteinuria, hemoglobin, and BMI.

All datasets preserve patient identifiers, enabling patient-level linkage between episodic clinical records and time-stamped wearable-like records.

#### 2.4.2 Time-stamped wearable-like records with Gretel.ai

Gretel.ai (37) was used to generate synthetic time-stamped wearable-like records with three daily readings, one every eight hours. The generated variables were aligned with the subset of parameters that could plausibly be obtained from repeated or near-continuous monitoring, including systolic and diastolic blood pressure, blood glucose, body temperature, and heart rate. The same patient identifiers were preserved to allow scenario-level linkage with Synthea consultation records.

The generation prompts were designed to preserve physiological plausibility and to introduce gestational-stage patterns compatible with pregnancy progression. For example, risk-oriented cohorts included patterns such as sustained hyperglycemia or elevated blood pressure. These generated records should be interpreted as simulated repeated-measurement records rather than as measurements collected from a commercial wearable device.

#### 2.4.3 Experimental methodology and reproducibility

The generation pipeline, available on GitHub (38), was designed to be auditable and reproducible. Configuration parameters, generation seeds, filtering scripts, and exported CSV files were organized to allow the same simulation workflow to be re-executed in a controlled computational environment. The Synthea and Gretel.ai outputs were subsequently used for DT inference and scenario visualization, not as replacements for real clinical validation.

#### 2.4.4 Dataset limitations

The synthetic datasets provide flexibility, privacy protection, and reproducibility, but they also introduce important limitations. The cohort size of n=500 is small for complex modelling, rare clinical events are underrepresented, and a single generation pipeline cannot reproduce the heterogeneity of real obstetric populations. Moreover, the time-stamped wearable-like records were generated under simplified physiological assumptions and do not capture the full range of sensor noise, adherence patterns, missingness, or high-resolution maternal physiology expected in real-world monitoring.

### 2.5 Prediction framework and machine learning models

Three supervised prediction tasks were defined: maternal risk stratification, GDM prediction, and preeclampsia risk estimation. Each task was trained and evaluated on its corresponding public development dataset. Synthea and Gretel.ai records were used only after model development for inference, scenario generation, and record-level DT summarization.

#### 2.5.1 Handling of time-stamped records at inference time

The wearable-like stream consists of time-stamped repeated measurements generated to emulate continuous monitoring at the data-acquisition layer. In the current proof-of-concept, however, the prediction modules are snapshot-based classifiers rather than temporal sequence models. Each clinical encounter or wearable-like measurement is processed as an independent physiological record at inference time. Timestamps are preserved for ordering, patient-level linkage, and gestational-stage summarization, but they are not used as input predictors and temporal dependencies are not modelled.

The wearable-like stream was not reduced to the most recent measurement. All available time-stamped records were processed record by record after model training. Model development was also performed at record level: the public datasets provide tabular observations or predefined record-level splits, not validated patient-level sequences of prenatal visits. Thus, the translation from model development to high-frequency DT simulation is based on repeated record-level inference, not on temporal resampling, sequence modelling, or extrapolation from a single time point. Table 3 summarizes this distinction.

**Table 3:** Handling of clinical and wearable-like records in the digital twin simulation.

Data stream	Temporal unit	Model handling	Use in DT simulation
Clinical data	Prenatal consultation-level record	Each encounter is processed as an independent physiological snapshot.	Used for post-training inference and patient-level scenario interpretation.
Wearable-like data	Time-stamped repeated measurement, generated every eight hours	Each measurement is processed as an independent physiological snapshot; no sequence model is trained.	Used for post-training inference, patient-level linkage, and gestational-stage summarization.

The model-selection strategy was task-specific. The maternal risk task included an internal comparison of candidate classifiers. The GDM module used RF as a supported tabular-learning baseline for heterogeneous clinical variables (39–41). The preeclampsia module used MLP models as exploratory nonlinear classifiers, consistent with comparative work in preeclampsia-related prediction (42, 43). The aim was not to perform an exhaustive benchmark for every condition, but to implement condition-specific modules for downstream inference and DT scenario generation.

The system processes two complementary input sources:

- **Wearable-like data.** Synthetic time-stamped wearable-like records containing variables that could plausibly be monitored frequently, including systolic and diastolic blood pressure, blood glucose, body temperature, and heart rate. These records contain three measurements per day.
- **Clinical data.** Episodic measurements obtained during simulated prenatal consultations, including demographic variables and laboratory markers when available.

Clinical and wearable-like data share some physiological variables but differ in temporal resolution and clinical richness. Accordingly, the framework was not formulated as a single feature-level multimodal classifier. Instead, both streams were processed separately and combined at the interpretation stage to compare their behaviour and to produce a unified risk summary.

#### 2.5.2 Overall prediction pipeline

**Figure 2:**
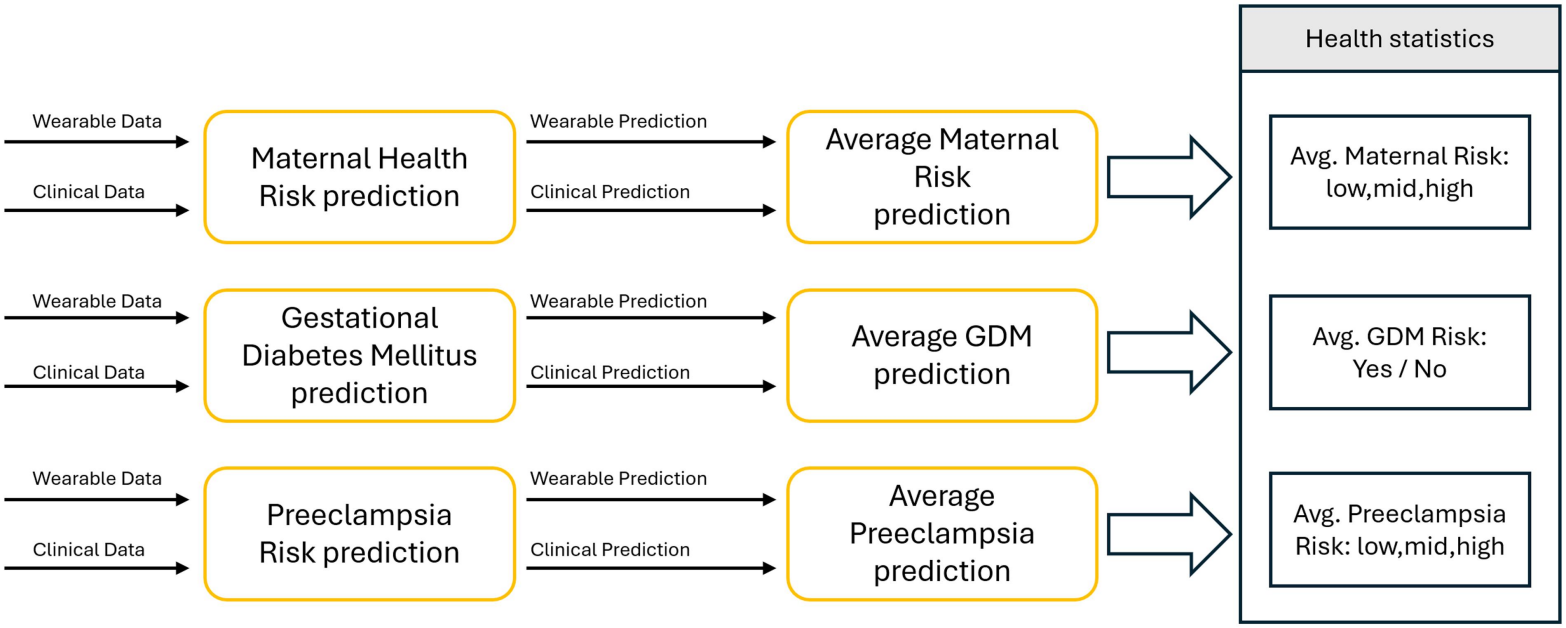
Prediction workflow of the proposed framework based on clinical and wearable data streams.

Figure 2 summarizes the prediction workflow. Three prediction tasks were considered: maternal risk, GDM, and preeclampsia. For each task, the available predictors depended on both the source of information and the clinical relevance of the target condition. This distinction is particularly important in preeclampsia prediction, where proteinuria and hemoglobin are clinically relevant but not available from wearable-like monitoring.

#### 2.5.3 Maternal risk prediction model

Maternal risk prediction was formulated as a multiclass classification problem with three labels: low, mid, and high risk. The model was developed using the Maternal Health Risk public dataset (32). The variables considered for model development are summarized in Table 4.

**Table 4:** Variables used for maternal risk model development.

Variable	Description
Age	Age of the woman during pregnancy in years.
Systolic BP	Upper value of blood pressure in mmHg.
Diastolic BP	Lower value of blood pressure in mmHg.
BS	Blood glucose level in the source dataset, expressed in mmol/L; values were converted to mg/dL for cross-source visual comparison when required.
Heart Rate	Resting heart rate, measured in beats per minute.
Risk Level	Target variable representing pregnancy risk classified as low, mid, or high risk.

BP, blood pressure; BS, blood sugar.

All variables were used as input features for model development, and *Risk Level* was used as the target class. K-Nearest Neighbors (KNN), Support Vector Machines (SVM), Random Forest (RF), and Extreme Gradient Boosting (XGB) were evaluated.

#### 2.5.4 Gestational diabetes prediction model

GDM prediction was implemented using RF classifiers and the Gestational Diabetes Dataset (33). The dataset contains metabolic, demographic, obstetric, clinical, and lifestyle-related variables associated with GDM, summarized in Table 5. RF was used as the final classifier because previous GDM studies using the same feature structure or closely related Kaggle-based tabular GDM datasets reported strong or competitive RF-based performance when compared with other machine learning models (39–41). Therefore, in this module, RF was adopted as a previously supported tabular-learning baseline for downstream inference and scenario generation, rather than as the result of a new exhaustive benchmark within this study.

**Table 5:** Variables analyzed from the gestational diabetes dataset.

Variable	Description
Age	Age of the patient.
No. of Pregnancy	Number of previous pregnancies.
Gestation in Previous Pregnancy	Indicates whether there was a gestation in previous pregnancies (Yes/No).
BMI	Body mass index.
HDL	HDL cholesterol level (high-density lipoproteins).
Family History	Indicates whether there is a family history of diabetes (1-Yes, 0-No).
Unexplained Prenatal Loss	Unexplained prenatal loss (1-Yes, 0-No).
Large Child at Birth	Indicates whether there was a large baby at birth.
PCOS	Presence of polycystic ovary syndrome.
Systolic BP	Systolic blood pressure in mmHg.
Diastolic BP	Diastolic blood pressure in mmHg.
OGTT	Oral glucose tolerance test, measured in mg/dL.
Hemoglobin	Hemoglobin levels in g/dL.
Sedentary Lifestyle	Indicates whether the patient has a sedentary lifestyle (Yes/No).
Prediabetes	Indicates whether the patient had prediabetes before pregnancy.
Class Label	Target variable indicating whether the patient develops gestational diabetes.

Two feature configurations were considered to reflect the difference between clinical and wearable-like monitoring. The clinical configuration retained variables available from consultation or laboratory assessment, including age, BMI, systolic and diastolic blood pressure, and glucose-related measurements such as OGTT. The wearable-like configuration was restricted to variables plausibly available through repeated monitoring in this framework, namely blood glucose and systolic/diastolic blood pressure. Continuous variables were normalized and scaled before model development.

#### 2.5.5 Preeclampsia prediction model

Preeclampsia prediction was formulated as a three-class problem using the *Preeclampsia in pregnant women* public dataset (34) and the reference notebook described in Wazir (44). MLP models were selected because preeclampsia risk estimation may involve nonlinear interactions among maternal characteristics, blood pressure, obstetric history, and laboratory markers. This choice is supported by previous comparative work in preeclampsia-related prediction, where MLP showed strong discriminative performance and ranked among the best-performing models together with SVM and RF (42, 43). The source dataset provides a predefined train/test split, which was used to compute the performance metrics reported in the Results section. Synthetic Synthea and Gretel.ai records were not used to train or test the MLP models; they were used only afterwards to generate framework-level inference scenarios.

Two MLP variants were developed. The wearable-feature model used only systolic and diastolic blood pressure, because these were the preeclampsia-related variables available in the synthetic wearable-like stream. The clinical-feature model incorporated systolic and diastolic blood pressure together with age, BMI, hemoglobin, and proteinuria. This separation was designed to test how much predictive information is lost when the model is restricted to hemodynamic variables only.

The MLP architecture consisted of fully connected layers with dropout regularization and softmax output for the three risk classes. The model was trained with categorical cross-entropy loss and the Adam optimizer. Because the available dataset is small and imbalanced, the model outputs were interpreted as exploratory risk stratification results rather than clinically deployable predictions.

## 3 Results

The Results section follows the three functions defined in the Introduction. First, Tables 7, 9, and 10 report model behaviour on the corresponding development datasets. Second, the scenario analyses show how trained models are applied to synthetic clinical and wearable-like records after training, using repeated snapshot-level inference rather than temporal sequence modelling. Third, Table 11 brings the three module outputs together at the interpretation layer. Synthetic DT data are therefore used to examine workflow behaviour and scenario consistency, not to provide clinical evidence of diagnostic validity.

### 3.1 Maternal risk classification and prediction

The first task evaluates whether the framework can provide a general maternal risk signal from routinely available physiological variables. This task is not intended to diagnose a specific disorder, but to provide a broad screening-oriented risk layer within the DT. The model classifies each record into low-, mid-, or high-risk categories using vital signs and demographic information.

Table 6 summarizes the candidate algorithms evaluated for this task and their optimized hyperparameters. The comparison was used to select the model that best fits the intended role of the DT as an interpretable screening component rather than as an isolated diagnostic classifier.

**Table 6:** Algorithms evaluated for maternal risk.

Model	Hyperparameters	Precision
KNN	K=19, weights = uniform, metric = Manhattan	0.660
SVM	class_weight = balanced, C=200, kernel = linear, degree = 5, coef0 = 1.0, gamma = auto, shrinking = True, probability = False	0.540
RF	bootstrap = True, criterion = entropy, max_depth = 75, max_features = 0.6, min_samples_leaf = 5, min_samples_split = 20, n_estimators = 50	0.690
XGB	n_estimators = 200, class_weight = balanced, learning_rate = 0.01, max_depth = 5, min_samples_split = 7, min_samples_leaf = 5, subsample = 0.6, max_features = None	0.720

Although Table 6 reports a slightly higher overall value for XGB than for RF, RF was selected because its per-class behaviour was more appropriate for the screening role envisaged in this proof-of-concept framework. In particular, RF provided stronger performance for the clinically more actionable low- and high-risk categories, whereas both RF and XGB performed less consistently in the intermediate class. In a screening context, failing to identify clearly high-risk cases is more consequential than optimizing the ambiguous mid-risk category. RF also provides feature-importance estimates, which supports transparency in clinical decision-support research.

Table 7 summarizes the RF performance. The model performs well for the low-risk and high-risk categories, but the mid-risk class shows low recall. This indicates that the intermediate category overlaps with adjacent classes in the available feature space and should be interpreted as a grey zone requiring clinical reassessment, not as a definitive standalone clinical state.

**Table 7:** Random forest performance for maternal risk.

Category	Precision	Recall	F1-score	Support
Low risk	0.73	0.99	0.84	70
Mid risk	1.00	0.12	0.22	32
High risk	0.81	0.88	0.85	34

#### 3.1.1 Post-training synthetic scenario inference

After model selection, the RF classifier was applied to synthetic clinical-consultation records and to time-stamped wearable-like records to inspect how the DT workflow handles both input streams after training. These analyses are reported as framework-level inference checks, not as additional clinical-performance estimates. Each synthetic clinical encounter or wearable-like measurement was processed independently using the same input-variable schema as the development dataset; timestamps were used only for ordering, patient-level linkage, and gestational-stage summarization.

The synthetic scenario records do not include independently validated longitudinal outcome labels. For this reason, maternal-risk scenario outputs are summarized by input stream rather than reported as calendar-based clinical outcome metrics. Table 8 summarizes the post-training inference outputs generated from each synthetic input stream and the interpretation assigned to those outputs within the proof-of-concept DT.

**Table 8:** Post-training maternal-risk inference outputs in synthetic DT scenarios.

Input stream	Inference unit	Output generated	Interpretation in this study
Synthetic clinical-consultation records	Consultation-level physiological snapshot	Record-level maternal-risk class generated by the trained RF model	Demonstrates post-training compatibility between the trained model and episodic clinical records; not used as evidence of real-world risk incidence.
Synthetic wearable-like records	Time-stamped physiological snapshot generated every eight hours	Repeated record-level maternal-risk classes generated by the trained RF model	Demonstrates that high-frequency input is handled as repeated snapshot-level inference; clinical interpretation requires aggregation, calibration, and patient-specific baselines.

Overall, the maternal risk results indicate that the selected RF model provides a screening-oriented baseline on the development dataset and that the DT workflow can process both episodic and repeated synthetic records after training. The synthetic scenario outputs are therefore interpreted as workflow-feasibility findings rather than as clinically validated longitudinal risk estimates.

### 3.2 Gestational diabetes classification and prediction

The second task evaluates GDM risk. Compared with the general maternal risk task, this prediction problem is more directly linked to measurable metabolic variables, particularly glucose-related information. Therefore, it is well suited to a DT monitoring framework in which clinical and wearable-like configurations are processed separately and compared at the interpretation stage.

Table 9 summarizes the Random Forest performance obtained for the two GDM input configurations. The clinical-data configuration uses episodic consultation-like variables, whereas the wearable-like configuration uses variables that could plausibly be monitored more frequently, including glucose-related measurements and hemodynamic information. The same class definitions are used in both configurations, but the input streams differ in clinical richness and temporal resolution.

**Table 9:** Random forest performance for GDM prediction under clinical-data and wearable-like configurations.

Input configuration	Category	Precision	Recall	F1-score	Support
Clinical data	No GDM	0.98	0.87	0.92	448
Clinical data	GDM	0.81	0.96	0.88	257
Wearable-like data	No GDM	0.97	0.88	0.92	448
Wearable-like data	GDM	0.82	0.95	0.88	257

Both configurations showed a similar behaviour. The model achieved high recall for the GDM class in both cases, indicating that most GDM-positive records in the development dataset were correctly identified. At the same time, precision was higher for the non-GDM class, suggesting that the selected metabolic and hemodynamic variables are informative for separating non-GDM and GDM profiles, although some false positives remain. The similarity between both configurations supports the internal consistency of the proposed DT workflow: when the target condition is strongly linked to a measurable physiological variable such as glucose, both clinical and wearable-like streams can provide coherent GDM-related risk estimates.

The trained model was also applied to synthetic scenario records representing pregnant patients undergoing treatment for prediabetes. Most of these patients were classified as not currently at risk for GDM. This result is plausible if treatment keeps observed glucose-related values within controlled ranges. However, it also exposes a limitation of the present implementation: treatment status and longitudinal medical history are not explicitly encoded as model inputs. Consequently, a patient with controlled prediabetes may appear similar to a low-risk metabolic profile if only current measurements are considered.

Overall, the GDM results represent the clearest positive finding of the proof of concept. Nevertheless, these results do not imply that wearable-like monitoring can replace clinical testing. GDM diagnosis and management require clinical context, laboratory confirmation, and professional interpretation.

### 3.3 Preeclampsia classification and prediction

The third task evaluates preeclampsia risk. This task is the most demanding because preeclampsia is not defined by blood pressure alone; clinical biomarkers such as proteinuria and additional maternal characteristics are central to risk stratification. For this reason, two MLP models were evaluated: one restricted to wearable-accessible variables and one using clinical variables. The purpose of this comparison is not to claim clinical readiness, but to quantify how much information is lost when preeclampsia monitoring is reduced to blood pressure alone.

Table 10 summarizes the performance of both MLP configurations. The wearable-feature model uses only systolic and diastolic blood pressure, whereas the clinical-feature model additionally incorporates clinical variables such as age, BMI, hemoglobin, and proteinuria. The same three risk categories are used in both configurations, but the two models differ in the amount of clinical context available at inference time.

**Table 10:** MLP performance for preeclampsia risk under wearable-feature and clinical-feature configurations.

Input configuration	Category	Precision	Recall	F1-score	Support
Clinical data	Low risk	0.50	0.82	0.62	11
Clinical data	Mid risk	1.00	0.76	0.86	25
Clinical data	High risk	0.50	0.40	0.44	5
Wearable-like data	Low risk	0.54	0.64	0.58	11
Wearable-like data	Mid risk	0.88	0.88	0.88	25
Wearable-like data	High risk	0.33	0.20	0.25	5

The combined results show a clear difference between the two configurations. The wearable-feature model performs best for the mid-risk class, but its performance for the high-risk class is weak. In particular, the high-risk recall of 0.20 indicates that most high-risk cases in the test set were not detected when only blood pressure variables were available. This is a critical limitation for any screening application and shows that the wearable-feature model is not suitable as a standalone preeclampsia predictor.

When clinical variables are included, the high-risk profile improves: high-risk recall increases from 0.20 to 0.40 and the high-risk F1-score increases from 0.25 to 0.44. This improvement is consistent with the inclusion of variables that are clinically relevant to preeclampsia risk assessment, such as age, BMI, hemoglobin, and proteinuria. However, the high-risk recall remains limited even in the clinical-feature configuration, so the model should still be interpreted as exploratory rather than clinically deployable.

**Figure 3:**
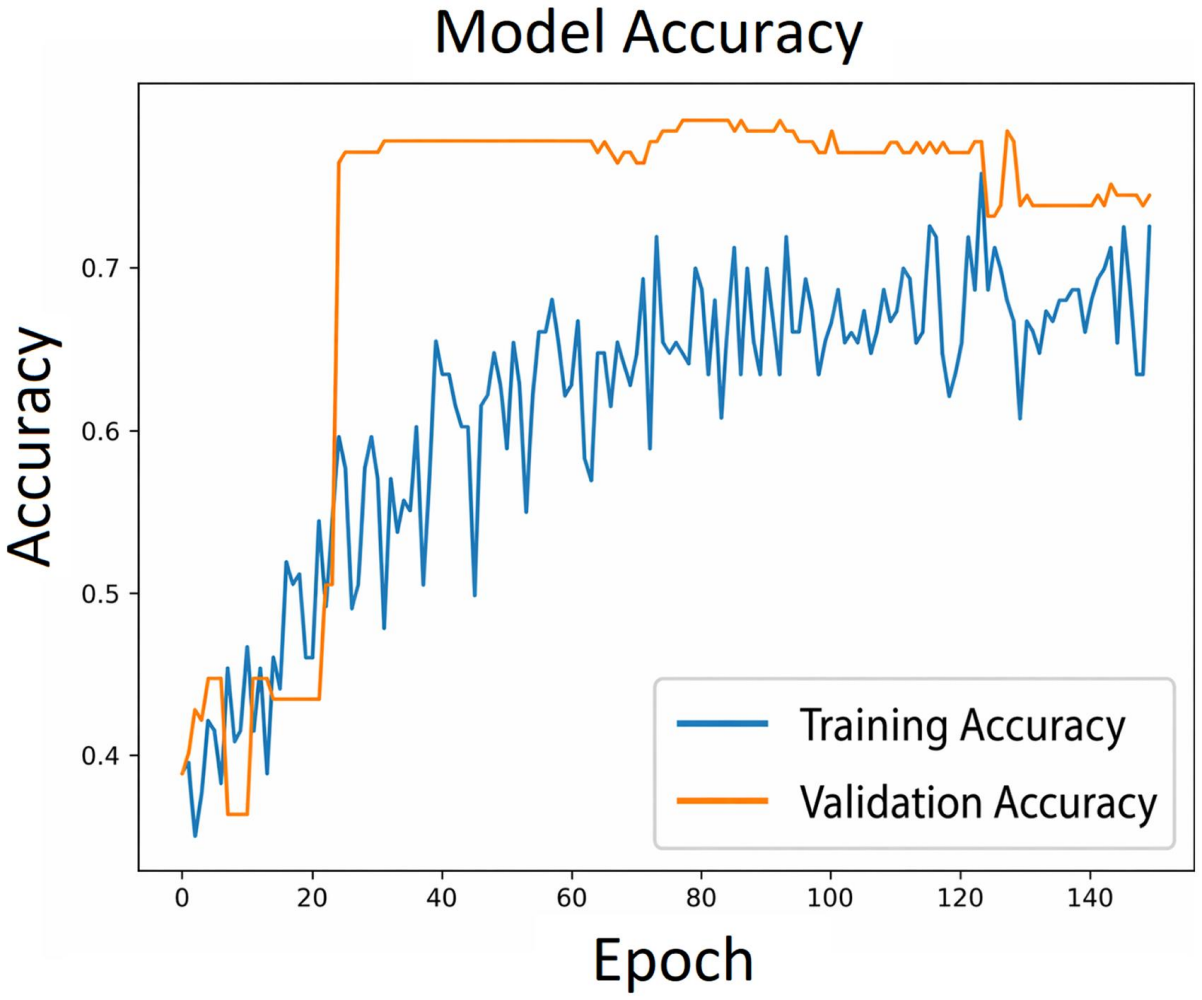
Training curve preeclampsia (wearable data).

Figure 3 shows the training curve for the wearable-feature model. The validation curve reaches a plateau, suggesting that simply increasing the number of epochs would not address the main limitation. The limiting factor is the restricted feature set and the small, imbalanced nature of the available data, rather than training duration alone.

Overall, the comparison in Table 10 is central to the interpretation of the preeclampsia module. Blood pressure provides useful hemodynamic information, but it does not capture proteinuria, hemoglobin status, BMI, age-related context, or prior medical history. Therefore, wearable-only preeclampsia prediction should be understood as an exploratory monitoring signal, not as reliable risk stratification.

#### 3.3.1 Clinical scenario analysis

The clinical-feature model was then applied to synthetic DT scenario datasets representing healthy pregnancies, pregnancies under hypertension treatment, and pregnancies with severe untreated hypertension. These analyses evaluate qualitative behaviour of the DT workflow rather than estimating real-world preeclampsia incidence.

**Figure 4:**
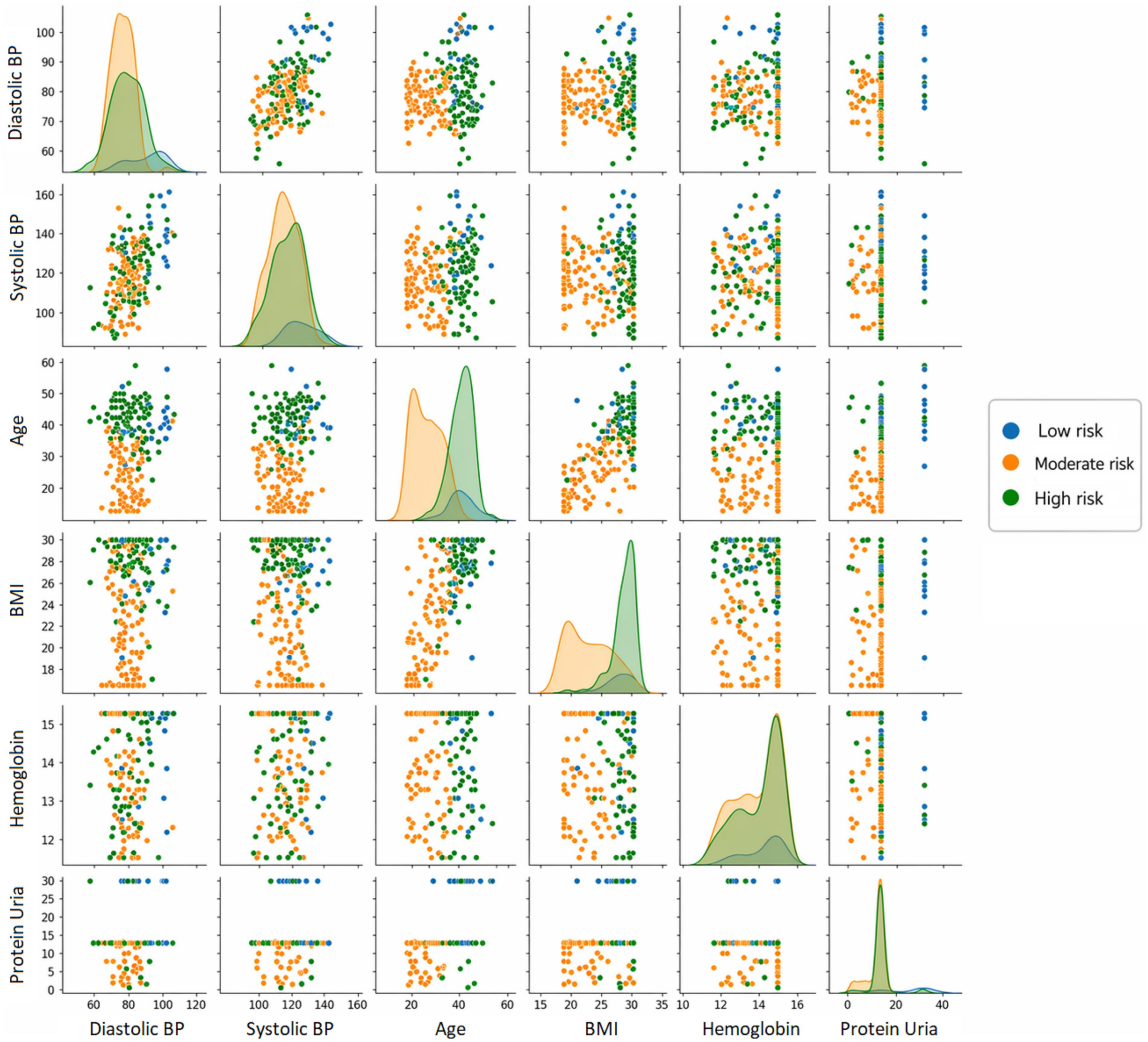
Scatter plot of relationships between factors and preeclampsia risk classes (clinical data).

Figure 4 shows the joint distribution of clinical variables and predicted classes. The model captures plausible associations between elevated blood pressure, BMI, age, proteinuria, and increased predicted risk. However, some healthy simulated patients are also classified as moderate or high risk when their feature distributions resemble higher-risk profiles. This finding indicates that the model may overestimate risk in certain scenarios and requires calibration before any clinical use.

**Figure 5:**
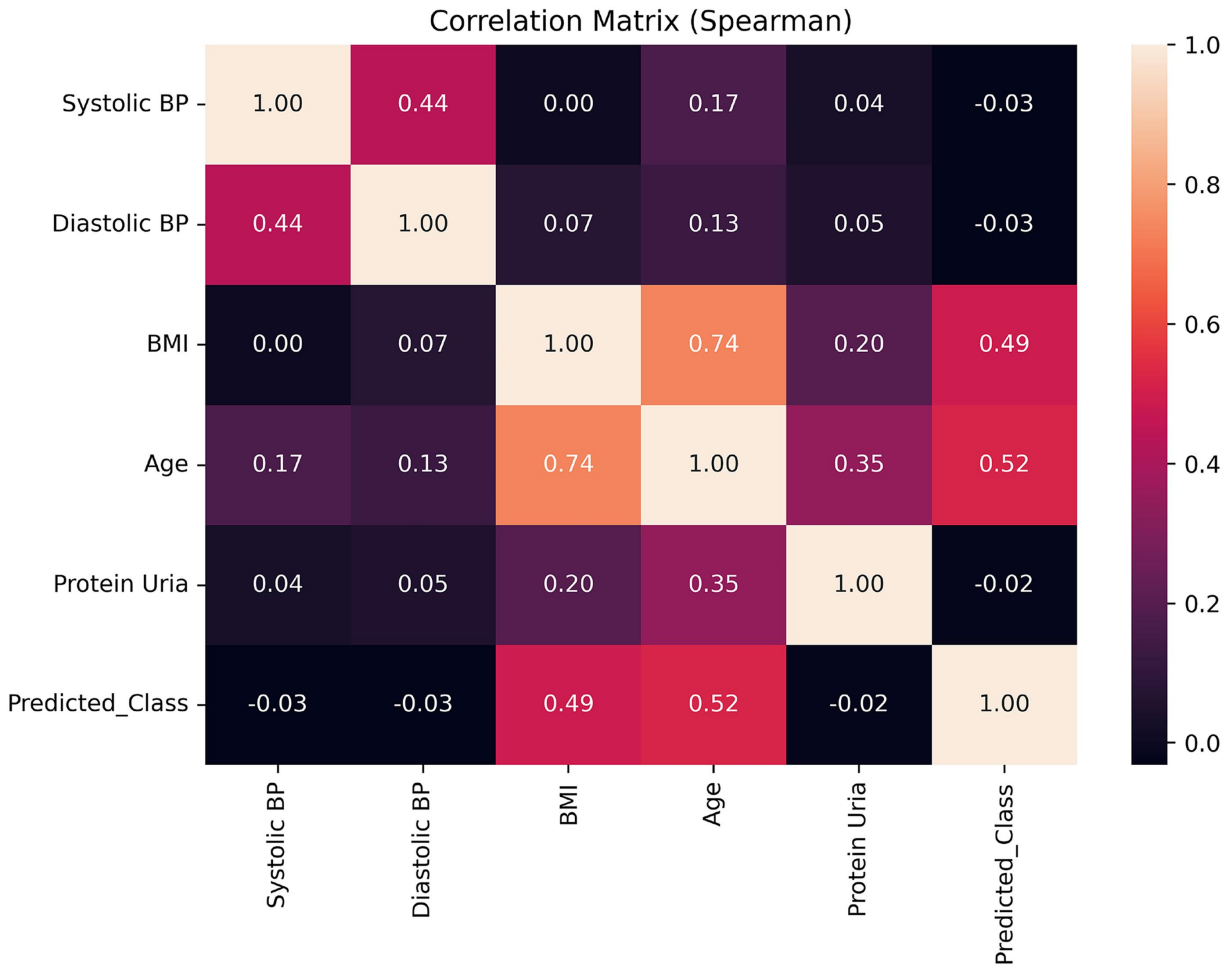
Spearman correlation matrix between clinical variables and predicted risk class.

Spearman correlation coefficients between the input variables and predicted risk class are shown in Figure 5. These correlations are descriptive and should be interpreted as an analysis of model-output behaviour in synthetic scenarios, not as evidence of causal relationships or real clinical associations.

**Figure 6:**
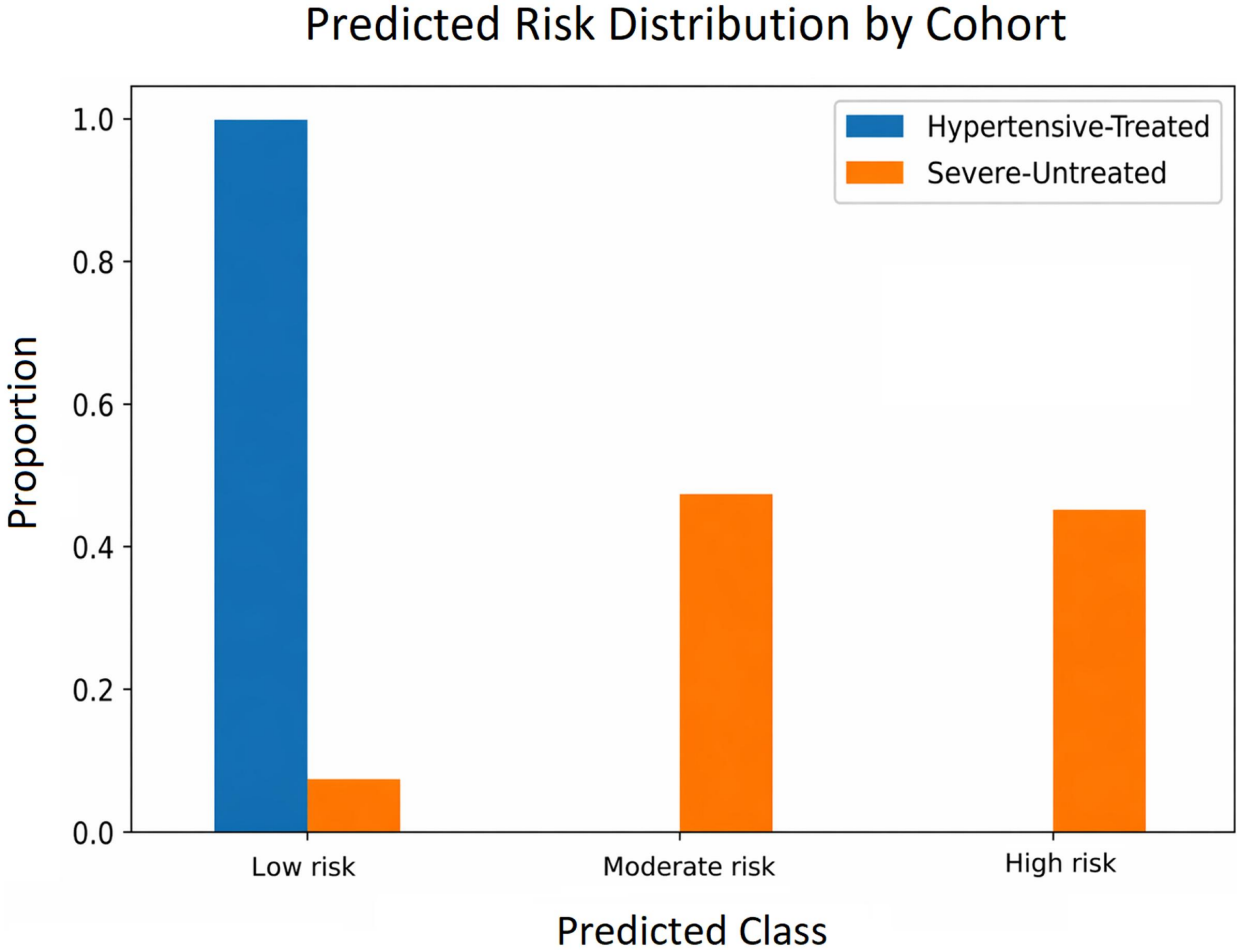
Predicted preeclampsia risk distribution by hypertension scenario.

Figure 6 compares treated hypertension and severe untreated hypertension scenarios. The model assigns lower risk to many treated cases, which is consistent with controlled observed measurements. However, this also demonstrates that treatment status and previous diagnoses should be explicitly represented in future models. In the severe untreated hypertension scenario, predictions shift toward higher-risk categories, showing that the model responds strongly to elevated blood pressure values.

#### 3.3.2 Wearable-like scenario analysis

**Figure 7:**
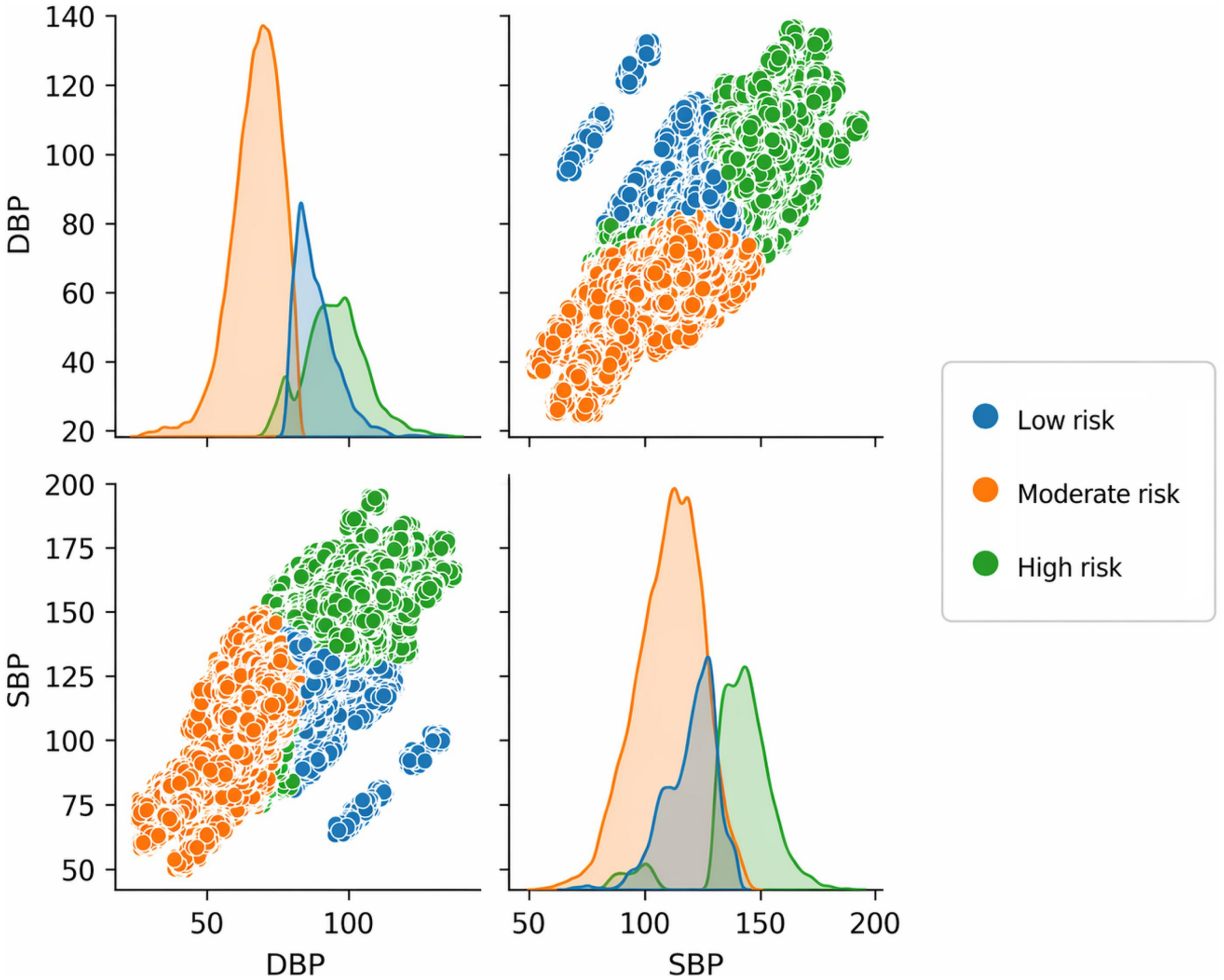
Relationships between variables by class in wearable data.

Finally, the wearable-feature model was applied to time-stamped wearable-like blood-pressure records. Most patients were classified as moderate risk. Figure 7 shows that the predictions follow a simple progression with systolic and diastolic blood pressure. This behaviour is expected because the model only has access to these two variables. The result reinforces the main finding of the preeclampsia analysis: repeated blood-pressure monitoring may be useful for surveillance, but it is insufficient for robust preeclampsia stratification unless complemented by clinical biomarkers and medical context.

### 3.4 End-of-pipeline digital twin output

The final step of the proposed DT workflow is an interpretation-level combination of the three prediction modules: general maternal risk, GDM risk, and preeclampsia risk. This combined output should not be interpreted as a new feature-level multimodal classifier. The three prediction tasks were developed using different benchmark datasets with different labels and feature spaces, and the synthetic DT scenarios do not provide a single ground-truth label for an overall maternal-health endpoint. Therefore, no additional global accuracy value is reported for a combined DT classifier. Instead, the end-of-pipeline result is a unified risk summary that brings together the outputs and limitations of the three modules for clinical interpretation.

Table 11 summarizes the end-of-pipeline DT output. It shows how the individual model results are combined at the reporting layer and what each module contributes to the final DT interpretation.

**Table 11:** End-of-pipeline combined digital twin output summary.

DT output field	Evidence from module results	End-of-pipeline interpretation	Clinical-use constraint
General maternal risk	The RF model performed better for low-risk and high-risk classes than for the mid-risk class.	Provides a broad screening-oriented risk signal; low- and high-risk outputs are more informative than the intermediate class.	Mid-risk predictions should be treated as a grey zone requiring clinical reassessment.
GDM risk	The RF model achieved high recall for the GDM class in both clinical and wearable-like configurations.	Provides the most stable module-level signal because glucose-related variables are available in both input streams.	Does not replace laboratory confirmation or clinical diagnosis of GDM.
Preeclampsia risk	The wearable-feature MLP showed weak high-risk detection; the clinical-feature MLP improved the profile but remained limited.	Provides an exploratory surveillance signal and highlights the need for clinical biomarkers.	Wearable-accessible blood pressure alone is insufficient for robust preeclampsia risk stratification.
Unified DT summary	The three modules are reported together after separate model inference.	Produces a structured patient-level risk summary for DT visualization and decision-support research.	The summary is not an autonomous diagnosis and must be interpreted under medical supervision.

The end-of-pipeline output is therefore a transparent reporting layer that identifies where the framework is informative, such as GDM-related monitoring and broad maternal risk screening, and where it remains insufficient, particularly wearable-only preeclampsia risk estimation. This design is consistent with the proof-of-concept scope of the study: the DT organizes heterogeneous model outputs into a unified clinical summary, but it does not yet provide a validated end-to-end clinical decision-support score.

### 3.5 Summary of proof-of-concept findings

Across the three prediction tasks and the end-of-pipeline summary in Table 11, the DT framework behaved differently depending on the clinical nature of the target condition and the variables available in each data stream. Maternal risk prediction showed that the framework can summarize snapshot-level predictions across gestational stages, but also that static classifiers trained on episodic records require calibration before being applied to repeated wearable-like data. GDM prediction showed the most stable behaviour, because glucose-related variables provide a direct physiological signal that can be represented in both clinical and wearable-like streams. Preeclampsia prediction produced the clearest limitation: blood-pressure-only wearable monitoring is not sufficient for robust risk stratification, and clinical biomarkers such as proteinuria and hemoglobin remain necessary.

These findings define the contribution of the paper as a DT proof of concept. The framework is not presented as a validated clinical predictor. Rather, it demonstrates how heterogeneous data sources, model-development datasets, and synthetic time-stamped scenarios can be organized into a reproducible perinatal DT workflow. The results support the value of the DT as an integration and visualization architecture, while also identifying the conditions under which wearable-like monitoring alone is insufficient and clinical interpretation remains essential.

## 4 Discussion

The findings should be interpreted against the three functions established in the Introduction: developing condition-specific prediction modules, applying them to synthetic clinical and wearable-like scenarios after training, and combining their outputs at the DT interpretation layer. Taken together, the results support the methodological value of the proposed architecture, but they also define clear boundaries for what the current proof of concept can claim.

The first function was to evaluate whether separate prediction modules could provide usable risk signals for the DT workflow. The maternal-risk model was useful mainly as a screening-oriented component: RF performed well for low- and high-risk cases, while the mid-risk class remained a grey zone requiring clinical reassessment. The GDM module produced the most stable results because glucose-related variables are directly linked to the target condition and can be represented in both clinical and wearable-like configurations. By contrast, the preeclampsia module exposed the strongest limitation of wearable-only monitoring: blood pressure is clinically relevant, but it is insufficient to represent preeclampsia risk reliably without proteinuria, laboratory markers, BMI, age, and medical history.

The second function was to examine post-training DT scenario inference. This step showed that episodic clinical records and repeated wearable-like records can be processed within the same workflow, but not with the same clinical meaning. Clinical records are less frequent but richer in contextual and laboratory information, whereas wearable-like records provide higher sampling density with a narrower feature set. The current models therefore operate as repeated snapshot-level classifiers. Future implementations should incorporate calibrated temporal summaries, uncertainty estimates, and patient-specific baselines before high-frequency monitoring outputs are interpreted clinically.

The third function was to bring the module outputs together. The end-of-pipeline DT summary is an interpretation-level reporting layer, not an additional multimodal classifier trained on a shared end-to-end ground truth. This distinction is essential because the three prediction tasks use different datasets, labels, and feature spaces. The unified summary is useful for structuring patient-level information and highlighting where clinical interpretation is needed, but it is not an autonomous diagnosis or a validated decision-support score.

Several limitations follow directly from this scope. The DT scenarios were generated using synthetic data only, and no real patient records or real wearable signals were used. The model-development datasets are limited in size, class balance, and representativeness. The simulation does not fully capture missingness, adherence, sensor noise, treatment effects, or rare obstetric events. Finally, the current models should be interpreted as exploratory components for evaluating workflow feasibility, not as diagnostic tools.

Overall, the study shows how public datasets, synthetic clinical records, and time-stamped wearable-like records can be organized into a reproducible perinatal DT pipeline. Its main value lies in making the data roles, model boundaries, and interpretation layer explicit. The framework appears most promising for GDM-related monitoring and broad maternal-risk screening, while preeclampsia risk estimation requires richer clinical variables and real longitudinal validation before clinical deployment.

## 5 Conclusion

This work proposes and evaluates a proof-of-concept DT framework for pregnancy monitoring and early risk exploration. The framework integrates public model-development datasets with synthetic clinical and wearable-like records to simulate how maternal risk, GDM risk, and preeclampsia risk could be processed within a unified perinatal monitoring architecture.

The results show that the framework can organize heterogeneous data streams and generate interpretable time-stamped scenario summaries. Maternal risk and GDM models showed usable screening-oriented behaviour within the available datasets, whereas the preeclampsia models revealed important limitations, particularly when restricted to wearable-accessible blood pressure variables. This finding reinforces the need to combine repeated wearable-like monitoring with clinical biomarkers and medical interpretation.

Overall, the study provides a methodological basis for future DT-based maternal health systems. Before any clinical use, the framework must be validated with real longitudinal cohorts, expanded with richer clinical features, calibrated for patient-specific baselines, and evaluated in collaboration with healthcare professionals. The current implementation should therefore be understood as a reproducible research prototype and not as a clinically validated decision-support system.

## Data Availability

The original contributions presented in the study are included in the article/Supplementary Material, further inquiries can be directed to the corresponding author/s.
